# A protocol for a qualitative synthesis exploring people with stroke, family members, caregivers and healthcare professionals experiences of early supported discharge (ESD) after stroke

**DOI:** 10.12688/hrbopenres.13158.1

**Published:** 2020-10-29

**Authors:** Elaine O Connor, Eamon Dolan, Frances Horgan, Katie Robinson, Rose Galvin

**Affiliations:** 1 School of Allied Health, University of Limerick, Castletroy, Limerick, Ireland; 2 Connolly Hospital, Blanchardstown, Dublin, Ireland; 3 School of Physiotherapy, Royal College of Surgeons in Ireland, Dublin 2, Ireland; 4 Ageing Research Centre, Health Research Institute, University of Limerick, Castletroy, Limerick, Ireland

**Keywords:** Stroke rehabilitation, stroke, family members, healthcare professionals, early supported discharge, thematic synthesis, systematic review, qualitative research

## Abstract

Early supported discharge (ESD) facilitates a person with a stroke to be discharged from the acute hospital environment earlier than conventional care to continue their rehabilitation within the home with members of the multi-disciplinary team. A number of quantitative studies have highlighted benefits of ESD including a reduction in the length of inpatient stay, cost savings, as well as reducing long term dependency. This systematic review and qualitative synthesis explores the perspectives and experiences of those involved in ESD including people with stroke, family members, caregivers as well as the healthcare professionals involved in the delivery of the service.

A comprehensive literature search will be completed in the following databases CINAHL, PubMed Central, Embase, Medline, PsycINFO, Sage, Academic Search Complete, Directory of Open Access Journals, The Cochrane Library, PsycARTICLES and Scopus. Qualitative or mixed methods studies that include qualitative data on the perspectives and experiences of people with stroke, family members, caregivers and healthcare professionals of an ESD service will be included.

Methodological quality will be appraised using the ten-item Critical Appraisal Skills Programme checklist for qualitative research by two independent reviewers with a third reviewer involved should differences of opinion arise. Findings will be synthesised using thematic synthesis.

It is anticipated that the qualitative synthesis will provide a deeper understanding of the experiences of ESD which may serve to inform practice as well as assist in the development of new ESD services.

**PROSPERO registration:** CRD42020135197 – 28/04/2020

## Introduction

Approximately one in six people worldwide will have a stroke during their life with costs associated with hospital care as well as for care following discharge from the hospital ( Sunnerhagen *et al*., 2013). Within high income countries stroke is a major cause of disability adjusted life years lost ( Nordin *et al*., 2015). Whilst there has been an increase in the number of people having a stroke, survival rates have increased with improved secondary prevention ( Lee *et al*., 2011). There is evidence to suggest that recovery following a stroke is enhanced with the utilisation of stroke specialist services such as stroke units, hyperacute stroke units, community rehabilitation as well as multi-disciplinary health care teams ( Clarke & Forster, 2015; Csiba & Farkas, 2009; Rodgers & Price, 2017; Walker *et al.*, 2013). With the increasing prevalence of stroke, it is imperative that sustainable long term support after specialist discharge takes place ( Aziz *et al*., 2016).

Following stroke, the patient rehabilitation pathway within the hospital environment may vary and experiences of the service can be complex and multi-faceted ( ESRI & RCSI, 2014; Morris *et al.*, 2007). Early supported discharge (ESD) encompasses a form of rehabilitation for people with mild to moderate stroke that accelerates their discharge from the acute setting to the home environment in order to continue their rehabilitation as an alternative to conventional care ( Langhorne, 1995; Mas & Inzitari, 2015; Rodgers *et al.*, 1997; Teasell *et al.*, 2009). As ESD rehabilitation largely takes place within the person’s home, goals set are more realistic and more relevant to the person with stroke as well as being person-centred ( Bushnell *et al.*, 2018; Cowles *et al.*, 2017; Langhorne & Widen-Holmqvist, 2007; Sunnerhagen *et al.*, 2013). The recent National Institute for Health and Care Excellence (NICE) guidelines recommend that the ESD team should be multi-disciplinary with the rehabilitation at home consisting of an intensity equivalent to the rehabilitation received within an acute stroke unit ( NICE, 2019). ESD whilst internationally recognised as a model of best practice, has not been widely implemented within Ireland and England ( ESRI & RCSI, 2014; Fisher *et al.*, 2019). In conjunction to ESD, additional models of service delivery have emerged within the United Kingdom such as Reablement teams as well as Intermediate Care teams ( Scobbie *et al.*, 2015). Reablement is focused on a person being able to achieve independence before an assessment to determine the level of supports required on a long term basis whereas Intermediate Care is viewed as intensive rehabilitation of six weeks with a number of aims; to allow a person to return home following an admission to hospital, to prevent admission to a long term care setting as well to prevent an admission to hospital ( Northern Ireland, Department of Health, Social Services and Public Safety, 2014).

Several randomised controlled trials have examined the impact of ESD when compared to conventional or usual care on clinical, process or cost outcomes ( Donnelly *et al*., 2004; Indredavik *et al*., 2000 and Mayo *et al*., 2000). Langhorne & Baylan (2017) conducted a Cochrane review on ESD services for people with acute stroke which included 17 trials involving over 2,422 participants. Findings demonstrate that ESD resulted in a reduction in length of hospital stay ranging from 6–15 days ( Anderson *et al.*, 2000; Bautz-Holter *et al.*, 2002; Indredavik *et al.*, 2000; Mayo *et al.*, 2000; ) as well as reducing long term dependency and admission to residential care ( Langhorne & Baylan, 2017). Cost savings have also reduced as a result of ESD being implemented ( Brewer & Williams, 2010; Teng *et al.*, 2003; Tistad & von Koch, 2015; Xu *et al.*, 2018). The review also reported that ESD trials without co-ordinated multi-disciplinary team input in place, resulted in smaller effect sizes ( Langhorne & Baylan, 2017).

A number of primary qualitative research studies have also explored experiences and views of ESD from the perspective of persons with stroke, their family members, caregivers and healthcare professionals ( Chouliara *et al.*, 2014; Collins *et al.*, 2016; Lou *et al.*, 2017). It is imperative to synthesise findings pertaining to the experiences and perspectives of persons with stroke, family members, caregivers and healthcare professionals of ESD in order to inform practice as well as policy development. A previous qualitative review explored patients with stroke, caregiver and healthcare professionals understanding of ESD ( Osborne & Neville, 2019). This qualitative review will differ from Osborne & Neville’s (2019) review by expanding the number of databases searched and information sources, and conducting a thematic synthesis to generate descriptive and analytical themes. In the context of stroke rehabilitation, these synthesised findings will contribute to the understanding of the lived experience of ESD from a variety of stakeholder perspectives ( ESRI & RCSI, 2014; Fisher & Walker, 2011).

The primary aim of this synthesis is to explore the totality of evidence regarding the experiences and perspectives of persons with stroke, family members, caregivers and healthcare professionals of an ESD service.

## Methods

### Study design

By conducting a qualitative evidence synthesis, multiple perspectives can be brought together which may not be represented within a single study alone with the findings providing new and valuable knowledge to healthcare professionals ( Carroll, 2017; Sandelowski & Barrosso, 2007). Evidence generated from qualitative evidence synthesis is being increasingly used by the World Health Organisation to inform the “values, acceptability, equity and feasibility of its recommendations” ( Downe *et al.*, 2019, p.1). This review is registered with the International Prospective Register of Systematic Reviews (PROSPERO): ID number CRD42020135197 (28/04/2020).

### Search strategy

Multiple electronic sources will be searched and will include studies that have been restricted by year of publication as well as by language. For the purpose of the review, the years searched will be restricted to 1995–2020. In total, 11 databases will be accessed to determine relevant studies: CINAHL, PubMed Central, Embase, Medline, PsycINFO, Sage, Academic Search Complete, Directory of Open Access Journals, The Cochrane Library, PsycARTICLES and Scopus. Reference lists of included studies will also be searched to locate any additional studies. The search string for the databases to be used is available as Table 1. As “Early Supported Discharge” research emerged in 1995, literature searched will be restricted from 1995 to the present day. Key terms used are incorporated into the search strategy for example “early supported discharge”, “stroke”, “experiences” as well as utilising MeSH terms.

**Table 1.** Search strategy.

S1	Acute stroke OR stroke OR Cerebrovascular accidents OR CVA* OR Cerebrovascular disorders OR infarct OR incident stroke brain injury, chronic OR post stroke OR poststroke OR post-stroke OR cerebrovasc*
S2	Qualitative OR qualitative research OR experience OR experiences OR perception OR perceptions OR perspective OR perspectives OR lived experience OR interview* OR focus group* OR ethnograph* OR phenomenol* OR grounded theor* OR grounded-theor* OR narrative analysis OR ethnological research OR ethnomethodology*
S3	Early supported discharge OR early discharge service* OR Early supported hospital discharge OR ESD OR early discharge OR early OR earlier OR rapid discharge OR prompt OR accelerate* discharge OR acute OR subacute OR supported discharge OR transition of care OR Rehabilitation OR rehab OR rehabilitate OR home rehabilitation OR home therapy OR Patient discharge OR progressive patient care OR discharge OR home care services
S4	S1 + S2 + S3

### Study selection

**ESD Definition:** For the purpose of this synthesis, the definition of ESD will be considered in the context of each individual study but will broadly adhere to the principle of an accelerated discharge providing support and/or rehabilitation in the home from the acute setting among people with acute stroke ( Langhorne, 1995). Clarification will be sought from individual authors in cases where there is ambiguity over the ESD definition. Studies that are related to ESD services but are not pertaining to stroke will not be included.

**Population:** The population of interest will include persons with stroke, family members, caregivers as well as healthcare professionals of an ESD service.

**Study types:** Studies will be included that utilise qualitative study designs. Mixed methods studies where qualitative data can be extracted will also be included. All included studies will be in English and will have been peer reviewed. Theses will also be included. Excluded studies will include those that are quantitative, systematic reviews, protocols, opinion pieces, and editorials.

All studies will be imported into EndNote X9, a reference management system, to remove duplicates as well as studies not suitable for inclusion. Two reviewers (EOC and KR) will independently screen the titles and abstracts of all the articles generated from the database search to identify studies suitable for inclusion. A third reviewer (RG) will be consulted to screen the titles and abstracts if consensus cannot be reached. This ensures rigour during the screening phase as well as transparency during the decision making process ( Soilemezi & Linceviciute, 2018).

Full text articles will be screened by two reviewers (EOC and KR) in order to make final decisions around the inclusion of these however should there be any disagreement or uncertainty regarding the inclusion or exclusion of a study, the third reviewer (RG) will be consulted regarding the final decision of the inclusion of these full text articles.

A table outlining the inclusion and exclusion criteria of the included studies has been devised ( Table 2).

**Table 2.** Inclusion and exclusion criteria.

	Inclusion criteria	Exclusion criteria
**Sample**	Individuals deemed appropriate to engage in ESD Healthcare professionals Family members or carers All of the above must have direct experience of an Early Supported Discharge service	Individuals deemed unsuitable to engage in ESD Healthcare professionals Family members or carers All of the above that do not have direct experience of an Early Supported Discharge service
**Phenomenon** **of interest**	Individuals with stroke, carers, family members and healthcare professionals direct experience of an Early Supported Discharge service	Stroke related care and experiences of living with a stroke not associated with Early Supported Discharge
**Design**	Qualitative and mixed methods studies whereby the qualitative data can be extracted	Quantitative studies
**Evaluation**	Analysis of qualitative studies on lived experiences, thoughts, opinions of Early Supported Discharge following a stroke.	Studies whereby the methods are not described as qualitative
**Research type**	Publications from 1995 – 2020 Full text available in English Peer reviewed journal articles and thesis	Systematic reviews, protocols, opinion pieces, editorials

ESD - Early supported discharge

### Quality appraisal of the included studies

The Critical Appraisal Skills Programme (CASP) checklist for qualitative studies will be used to critically appraise the quality of the included studies ( CASP, 2018). The CASP checklist consists of 10 items which focus on three areas; are the results valid, what are the results and will the results help locally; that should be considered when appraising qualitative studies. The CASP checklist is succinct and allows for the quality of articles to be compared and contrasted ( Chenail, 2011; Nadelson & Nadelson, 2014). Included studies will be independently appraised using the CASP checklists by two reviewers (EOC and KR) with a third reviewer (RG) involved should any differences of opinions arise.

### Data extraction and synthesis

A table describing the descriptive characteristics of included studies will be developed. Information pertaining to authors, year of publication, setting (home, community, healthcare professionals’ workplace), methodology, participant group (persons with stroke, family member/carer or healthcare professionals), approach to data collection, method of analysis and key results/findings will be extracted.

Findings from articles included in this synthesis will be imported into NVivo Version 12 ( NVivo qualitative data analysis software [program], 2015). Benefits associated with the use of NVivo include efficiency and transparency ( Hoover & Koerber, 2010). A three stage process of thematic synthesis as described by Thomas & Harden (2008) will be undertaken. Firstly themes will be coded inductively –“line by line”, next – “descriptive themes” – will be generated and finally – “analytical themes” – will be developed ( Thomas & Harden, 2008). Thematic synthesis is an accessible form of synthesis that generates findings of direct relevance to policy and the design of interventions ( Barnett-Page & Thomas, 2009; Flemming *et al.*, 2019). The first author (EOC) will conduct the thematic synthesis; however, all three other authors (ED, KR and RG) will provide guidance and contribute to analytical discussions. All authors (EOC, ED, FH, KR and RG) will read and contribute to the final manuscript. 

In order to enhance trustworthiness and ensure rigour, the first author will engage in reflexive analysis. Reflexivity is an important element to research to ensure transparency as it involves critical self-reflection focusing on bias, preferences and preconceptions as well as the relationship to the research ( Korstjens & Moser, 2018). It is also important when engaging in reflexivity to review how both the researcher and participants impact on one another as well as the research ( Finlay, 2002). This will be achieved by maintaining a diary, engaging with supervisors and independent reviewers. In line with recommendations the lead author (EOC), an Occupational Therapist with clinical experience in stroke rehabilitation and Doctoral candidate, has been reflexive since the pre-research stage, reflecting on the research topic itself and their relationship to the topic ( Finlay, 2002).

### Dissemination of findings

On completion of the analysis, it is anticipated that the findings will be submitted for publication in a peer-reviewed journal. Findings will also be disseminated to persons with stroke, family members, healthcare professionals and policy makers and national stroke advocacy organisations such as the Irish Heart Foundation.

### Study status

This search is complete. 

## Discussion

This review will synthesise the evidence currently available as well as to add to the empirical evidence base in relation to stakeholders’ experiences and perspectives of ESD. It has been suggested that service user perspectives may be overshadowed by professional perspectives within the context of qualitative research therefore it would be important to ensure that the service user is at the forefront when developing and designing ESD services ( Berry & Hayward, 2011). In this review the perspectives of people with stroke, family members and healthcare professionals will all be considered and privileged. This review will contribute to the clinical and scientific understanding of ESD services by synthesising perspectives of multiple stakeholders as well as identifying gaps in the evidence base and areas for further research. This review will also generate findings of value to healthcare professionals, policy makers and others involved in the design and development of stroke rehabilitation services.

## Data availability

### Underlying data

No data are associated with this article.

### Reporting guidelines

Open Science Framework: PRISMA-P checklist for ‘A protocol for a qualitative synthesis exploring people with stroke, family members, caregivers and healthcare professionals experiences of early supported discharge (ESD) after stroke’ https://doi.org/10.17605/OSF.IO/A82RP ( O Connor, 2020).

Data are available under the terms of the Creative Commons Zero “No rights reserved” data waiver (CC0 1.0 Public domain dedication).
