# Supplementary material for: A protocol for a qualitative synthesis exploring people with stroke, family members, caregivers and healthcare professionals experiences of early supported discharge (ESD) after stroke
Source: HRB Open Res. 2020 Oct 29;3:79. [Version 1] doi: 10.12688/hrbopenres.13158.1 (PMC8185577; doi:10.12688/hrbopenres.13158.1)
Supplement: Peer review history

# Peer review: Reviewer response for version 1

**DOI:** 10.21956/hrbopenres.14279.r29495

**Published:** 2021-06-07

**Authors:** Elizabeth A Lynch

**Affiliations:** 1 Flinder University, Adelaide, Australia

This is a clearly presented protocol. Rationale is presented clearly, need for review and how this review will differ from the previously published review of qualitative studies is presented and justified. Planned methods clearly presented. 

 Quote re-incidence of stroke in first sentence is outdated - now estimated 1 in 4 people globally will have stroke - see Global, Regional, and Country-Specific Lifetime Risks of Stroke, 1990 and 2016 in NEJM 2020 ^1^.

 I wonder if it would be worth presenting how ESD differs from Rehab in the Home which is more common in some parts of the world - sometimes they have same structure as ESD programs, other times quite different - i.e. which RITH programs would be in, which would be excluded.

Is the study design appropriate for the research question?

Yes

Is the rationale for, and objectives of, the study clearly described?

Yes

Are sufficient details of the methods provided to allow replication by others?

Yes

Are the datasets clearly presented in a useable and accessible format?

Not applicable

Reviewer Expertise:

Stroke rehabilitation

I confirm that I have read this submission and believe that I have an appropriate level of expertise to confirm that it is of an acceptable scientific standard.

## References

[1] : Global, Regional, and Country-Specific Lifetime Risks of Stroke, 1990 and 2016. *New England Journal of Medicine* .2018;379(25) : 10.1056/NEJMoa1804492 2429-2437 10.1056/NEJMoa1804492 PMC624734630575491

# Peer review: Reviewer response for version 1

**DOI:** 10.21956/hrbopenres.14279.r28257

**Published:** 2020-11-02

**Authors:** Roman Romero-Ortuno

**Affiliations:** 1 Discipline of Medical Gerontology, Trinity College Dublin, Dublin, Ireland

This is a protocol for a qualitative synthesis of early supported discharge in stroke, which aims at incorporating the experiences of patients, family members, caregivers and healthcare professionals. The proposed search strategy and their aim to contact authors for further information are likely to address the objective to a very good extent. This may also lead to analysis of relevant 'grey literature'.

Is the study design appropriate for the research question?

Yes

Is the rationale for, and objectives of, the study clearly described?

Yes

Are sufficient details of the methods provided to allow replication by others?

Yes

Are the datasets clearly presented in a useable and accessible format?

Not applicable

Reviewer Expertise:

Geriatrics and Gerontology

I confirm that I have read this submission and believe that I have an appropriate level of expertise to confirm that it is of an acceptable scientific standard.
